# Evidence for Discriminant Specific Tastes in Chardonnay Wines Among Other White Wines

**DOI:** 10.3390/foods14162870

**Published:** 2025-08-19

**Authors:** Baptiste Seinforin, Soline Caillé, Maria Nikolantonaki, Cédric Saucier

**Affiliations:** 1 UMR Sciences pour l’Œnologie (SPO), University of Montpellier, INRAE, Institut Agro Montpellier, 2 Place Pierre Viala, 34000 Montpellier, France; baptiste.seinforin@umontpellier.fr (B.S.); soline.caille@inrae.fr (S.C.); 2 UMR Procédés Alimentaires et Microbiologiques (PAM), Institut Universitaire de la Vigne et du Vin-Jules Guyot, Institut Agro Dijon, INRAE, Université Bourgogne Europe, 2 Rue Claude Ladrey, 21000 Dijon, France; maria.nikolantonaki@u-bourgogne.fr

**Keywords:** sensory analysis, white wine, taste, Chardonnay, nose clip

## Abstract

The quality of white wine is related to sensory attributes like color, odor and taste. This study focused mainly on taste attributes of white wines. The research hypothesis was to find out if taste-related attributes alone, determined by sensory experiment, could discriminate Chardonnay versus non-Chardonnay wines. Sensory analyses were performed with a trained panel on commercial white wines made from single varieties. Black glasses and nose clips were used to remove sensory interference and to first assess only taste attributes. Initial tests were then performed to evaluate the possibility to discriminate against Chardonnay wines only due to taste. In a second series of experiments, Rate All That Apply (RATA) experiments were performed in a set of Chardonnay versus non-Chardonnay wines. An initial sensory experiment revealed that some of the Chardonnay wines could be discriminated against by taste only but that recognition by using olfaction was more powerful which confirmed our research hypothesis in part. The second series of RATA sensory analysis revealed that some specific descriptors such as fat, salt, bitter and acid are involved in the taste discrimination of Chardonnay versus non-Chardonnay wines, especially with Sauvignon Blanc wines. These findings suggest that while modal sensory approach remains more robust for varietal identification, taste alone offers some discriminatory power.

## 1. Introduction

Approximately 50% of the world’s vineyard area are dominated by only 16 grapevine cultivars [1]. Among these, *Vitis vinifera* cv. Chardonnay is notable for its extensive cultivation and remarkable phenotypic plasticity, particularly its resilience to climate change-related stressors such as increased temperature and drought [2]. Originating from the Burgundy region of France, Chardonnay is a spontaneous hybrid of Pinot Noir and Gouais Blanc, as demonstrated through microsatellite and SNP marker analyses [3]. Currently, it ranks as the fifth most extensively planted grapevine cultivar globally [4] and is the most widely planted white cultivar, having overtaken Airén, which historically held this status until approximately 2010 [1].

Global vineyard surface planted with Chardonnay were estimated at 210,000 hectares in 2017 [5], reflecting a 39% expansion over 17 years [1]. This cultivar’s broad adaptation is evidenced by its presence in over 41 countries, spanning diverse climatic zones from cool temperate to Mediterranean [6]. In France, the principal terroir of origin, Chardonnay occupies about 51,000 hectares, accounting for nearly 25% of worldwide acreage. While traditionally concentrated in Burgundy, viticultural expansion has led to larger plantings in the Occitanie region—19,000 ha versus 18,000 ha in Burgundy as of 2024—reflecting regional diversification in terroir and clonal selection [6].

Chardonnay demonstrates considerable terroir-driven phenotypic variation, including differences in berry skin thickness, phenolic compound accumulation, and acid metabolism, which translate into a diverse sensory profile. Cultivar versatility is reflected in its ability to express distinct chemical signatures influenced by soil composition, microclimate, and viticultural practices such as cluster thinning and canopy management. Despite this variability, Chardonnay wines typically exhibit an aromatic fingerprint characterized by volatile thiols, esters, lactones, and oak-derived compounds when barrel-aged [2]. Sensory descriptors commonly reported include diacetyl-associated buttery notes, vanillin, and toasted aromas resulting from oak maturation, alongside varietal markers such as floral (white flowers), fruity (citrus, green apple), and complex dried fruit notes, particularly in premium Burgundy expressions [7].

Although extensive research has documented the aromatic compounds contributing to Chardonnay’s varietal typicality, there remains a notable gap in understanding its gustatory profile independent of olfactory influence. Sensory studies to date have predominantly employed retronasal olfaction or combined aroma-taste evaluations, limiting insights into the pure taste components—such as bitterness, sweetness, acidity, and mouthfeel—that may contribute to cultivar identification. This study aims to address this gap by isolating gustatory perception through controlled sensory experiments, with the objectives of evaluating the ability of trained panelists to recognize Chardonnay wines based solely on taste and establishing a detailed gustatory vocabulary specific to this cultivar.

## 2. Materials and Methods

### 2.1. Panel and Ethic/Consent Data

The panel was composed of students on the Diplôme National d’Œnologue master’s degree program at the University of Montpellier. They were selected on a voluntary basis and signed an informed consent form to use anonymously their results. Fourteen panelists took part in the first part of the study on the recognition of Chardonnay (36% F/64% M) and twelve took part in the study of the descriptors (25% F/75% M). 

The French society of sensory analysts state that: “It being understood that sensory analysis studies aim to assess the perception or satisfaction of “healthy” consumers during consumption or during the actual or simulated use of products, these studies are not considered a priori as research on human subjects, and therefore do not fall within the scope of the law concerning research involving human subjects, i.e., governing research “organized and carried out on human beings with a view to developing biological or medical knowledge” (DGS-CPP of 22 March 2017).

Data collection followed the RGPD (Regulation on the protection of personal data) policy in France, panelist were informed about the use of their data and signed a consent form. They were also asked to make a commitment to good practice in the evaluation of alcoholic products.

A self-assessment provided by the Ethics Committee of INRAE was also performed to check the Ethical compliance of the study.

### 2.2. Wines

Commercial varietal wines were purchased or obtained as free samples from French appellations in Occitania or Burgundy to represent different appellations and terroirs. The wines selected, all single varietals were 10 Chardonnay (**C**) (IGP Pays d’Oc, IGP côtes de Thau, AOC Bourgogne, AOP Limoux Blanc, AOP Mâcon-villages), 5 Sauvignon B (**S**) (AOC Reuilly, IGP Val de Loire, IGP Pays d’Oc) and 5 Viognier (**V**) (IGP Pays d’Oc, IGP côtes de Thau). Information on these wines, including their alcohol content, is available in Appendix B.

### 2.3. Chardonnay Recognition

The training session was divided into two parts. During the first part, the panelists were invited to familiarize themselves with the tasting method used. To do this, 6 wines were tasted, with the grape variety presented, so that they could be trained to use a nose clip and think about identifying the grape variety being tasted. The panelist tasted 3 Chardonnays, 1 Sauvignon B and 2 Viogniers. During the second part of the session, the panelists tasted under the same conditions as those used for the subsequent evaluation sessions. First, the wine was served, then when everyone was served, the panelists put on their nose clips, tasted the wine and had to answer whether they thought the wine was a Chardonnay or not. Second, the panelists removed the nose clips and carried out an olfactory examination of the wine, answering the same question again.

### 2.4. Specific Taste Descriptors

Wines were analyzed using the Rate All That Apply (RATA) methodology [8]. During one session, the panelist were trained to exercise, understand and consistently use attributes (acidity, alcohol, bitterness, aqueous, fat, sweetness, short, long, salty and umami) and to familiarize themselves with the methodology. To help the panelist to identify and remember the sensory attributes standards were prepared by adding compounds to white wine (Appendix A, Table A1).

Wines were then analyzed over two tasting sessions. Analyses were conducted in individual testing booths. Samples (30 mL) were served at temperature 18.9 ± 1.3 °C in black glasses (to avoid any potential visual influence during evaluations), marked with random three-digit codes. Wines were presented in a monadic service, according to William’s Latin square design, to balance presentation order and carry-over effect [9]. Wines were analyzed in duplicate. For each sample, the panelist had to choose the most pertinent attributes, from the list of gustatory terms. Then, for each descriptor checked off, panelist rated its intensity on a 5-point scale from “low” on the left to “high” on the right. Data acquisition was assisted by FIZZ software (FIZZ network, v.2.518; Biosystème, Courtenon, France). 

### 2.5. Data Analysis

For the first part of this study, the rates of correct answers were collected and a binomial law distribution test was carried out to determine whether the answers were significant, i.e., considered not to have been chosen at random. The test was first applied to all the smell data, to the taste data, and finally from all the responses (taste + smell) to see if the wine was recognized overall or not and then to be able to compare the two smell and taste conditions. All unchecked descriptors were given a score of 0 and checked descriptors were given a score corresponding to the intensity chosen (between 1 and 5) [10]. Data obtained is therefore considered to be quantitative and their analysis was performed using XLSTAT software 2022.2.3.1288 (Lumivero, Paris, France).

Panel performance (discrimination, repeatability and consensus) was first checked with a three-way ANOVA (three factors: wine, panelist, repeat) and an analysis of their interactions. Then, a two-way ANOVA (two factors: grape variety, panelist) was run on the totality of the descriptors. Mean values for the grape variety were then compared through the Tukey multiple comparison test. The Dravnieks score is calculated for each product and each descriptor, and it is defined as the geometric mean of the percentage of panelist having ticked the descriptor and the percentage of the sum of the intensities chosen. This measure aims at balancing between the elicitation rate and the average score by those panelist that actually rated the product on the attribute under investigation [11,12]. Principal component analysis (PCA) type covariance and Ward’s method) were performed on the Dravnieks score.

## 3. Results

### 3.1. Chardonnay Taste Recognition

An initial analysis of the results by the panel revealed variability in the rates of grape variety recognition across the wines. To evaluate the panelist performance, a binomial test was applied to determine whether their choices deviated from randomness. The rates of correct answers and *p*-value associated are compiled in Table 1.

Nine out of the fourteen jurors showed statistically significant results (*p* < 0.05), indicating that the jury possessed discriminating ability for sensory analysis under the conditions used.

The recognition rates analysis showed higher overall performance for the nose (66.7%) compared to taste (58.6%), suggesting that olfaction is more effective for identifying grape varieties. This trend was reinforced by the binomial test results, which showed more significant outcomes for the olfactory modality.

To statistically compare gustatory and olfactory recognition rates, a paired *t*-test was conducted wine-by-wine. The test indicated a marginally significant trend favoring the nose (t = −1.75; *p* = 0.093), implying that olfaction provides better overall recognition of the wines. This finding is supported by a one-way ANOVA, which showed a non-significant but consistent difference (F = 2.30; *p* = 0.136).

Further analysis by grape variety revealed that Sauvignon B wines were the most accurately identified by taste as non-Chardonnay wines, with three out of five wines showing significant recognition (*p* < 0.05) and one nearing significance (*p* = 0.09). For Viognier, two out of five wines were significantly recognized as non-Chardonnay. Among the twelve Chardonnays tested, only one was significantly identified as Chardonnay, with two others approaching significance. It seems that Chardonnay wines have very different taste compared to Sauvignon B wines but that their taste could be close to Viognier ones. To better understand the different tastes involved a second series of sensory RATA experiments were performed with a selection of some of the wines that were better recognized as Chardonnay or non-Chardonnay (C1, C2, C3, C7, C9, C10, V1, V3, V4, S3, S4, S5) in the first series. These RATA experiments are described in the next section.

### 3.2. Specific White Wine Taste Descriptors by RATA Sensory Test

Interactions between wine and repetition and between wine and subject did not show any significant effect on sensory parameters, indicating that the panel repeatability and consensus were correct. Table 2 then allows to separately look at the effects and panelists and variety through an analysis of variance.

It is interesting to separately look at the effects of panelist andvariety. First, for all the descriptors, the panelist effect was highly significant (*p* < 0.0001). The grape variety effect was significant for several descriptors, including acidity (*p* < 0.0001), alcohol (*p* = 0.021), bitterness (*p* = 0.038) and fattiness (*p* < 0.0001). This suggests that these perceptions play a part in the taste identity of grape varieties, particularly Chardonnay, which is often perceived as being “fatter” or “rounder” on the palate.

**Figure 1:**
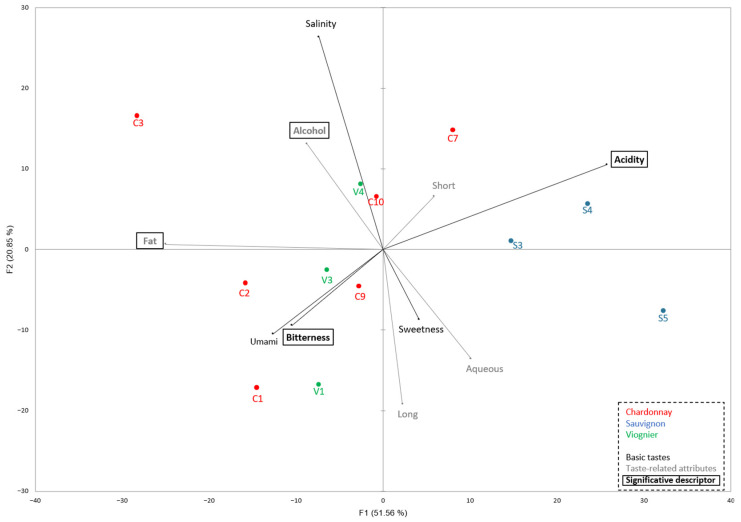
Principal component analysis of wine sensory profiles based on taste descriptors.

A Principal Component Analysis (PCA) was conducted to explore the relationships between sensory attributes and the different sample groups C, V, and S (Figure 1).

This PCA biplot reveals how samples are distributed in the multidimensional sensory space and how various sensory descriptors contribute to this distribution. The first two principal components (F1 and F2) account for a cumulative 72.41% of the total variance in the dataset (F1: 51.56%, F2: 20.85%). This high percentage suggests that the PCA provides a reliable two-dimensional representation of the sensory variability among the samples. The loading vectors (black arrows) represent sensory attributes. The direction and length of each vector indicate their correlation with the principal components: F1 (51.56%) appears to be primarily driven by Acidity, (positive side) versus Fatness and Bitterness (negative side). F2 (20.85%) differentiates samples based on Saltiness and Alcohol (positive axis) and to a lesser extent Long aftertaste (“length”, negative axis) and aqueoussness. The near-orthogonality of vectors such as Acidity and fattiness suggests low correlation between those traits, whereas vectors like bitterness and umami are closely aligned, indicating a strong positive correlation.

Distinct clustering patterns among the sample groups are evident. In detail, Sauvignon B samples (S3, S4, S5) are located on the positive side of F1 and are strongly associated with acidity, and a short aftertaste. This suggests that the Sauvignon B group possesses a lighter, more acidic sensory profile, which may be more refreshing or palatable depending on the product type. On the other hand, the Chardonnay group (C1–C10) shows greater dispersion, indicating internal heterogeneity. Indeed, C1 and C2 load negatively on F1 and F2, associated with fattiness and bitterness; while C3 is positioned far on the positive F2 axis and correlates strongly with saltiness and fattiness. C7 and C10 are located toward the quadrant representing a Short aftertaste and acidity, partially overlapping with Sauvignon B samples. Regarding the Viogner group, (V1–V4): V1 and V3 are similar to C1 and C2, clustering in the bitterness quadrant. V4, in contrast, is nearer to Alcohol and Saltiness, implying a different sensory character within this group.

## 4. Discussion

Our results reaffirm the important role of olfaction in wine perception, which is consistent with previous studies on the aromatic typicality of white grape varieties that is also linked to terroir [13]. However, some Sauvignon, Viognier, and Chardonnay samples could be identified by taste alone. This suggests the presence of specific taste-active molecules in certain wines that contribute to a characteristic varietal taste, depending on their concentration and perception thresholds.

Sauvignon wines were distinctly characterized by higher acidity compared to Chardonnay and Viognier in the RATA sensory experiments. They were also perceived with less mouthfeel structure compared to Viognier and Chardonnay with lower scores for the fat attribute (Figure 1). The differentiation between Viognier and Chardonnay was less pronounced. Chardonnay wines were perceived to be slightly saltier compared to Viognier (see Table 3). The differences in acidity, saltiness, bitterness, and fattiness mouthfeel between Sauvignon, Viognier, and Chardonnay wines are linked to their distinct compositions. These differences are primarily driven by phenolic content, pH levels, and the presence of other compounds such as polysaccharides and ethanol, which collectively shape the sensory profile of each grape variety [14,15,16,17]. Acidity is largely influenced by pH, affecting perceived freshness, Sauvignon B wines are known for their acidity [18]. Fat mouthfeel is modulated by ethanol and polysaccharides, which are more prominent in Chardonnay wines, contributing to its perceived roundness and viscosity [16,17]. Saltiness, though not widely studied in wine, may be indirectly shaped by mineral content and winemaking techniques, and could explain the slightly higher saltiness perceived in Chardonnay wines [19].

By analyzing taste descriptors without preconceived expectations, we were able to highlight specific attributes that warrant further investigation in future studies. While descriptors such as umami or salty helped discriminate certain white wine tastes, they were not effective for identifying the grape variety. Variety plays a key role in wine identification but the terroir, i.e., the impact of soil type, climate and viticultural practices, may shape the taste expression among varietal groups and will be investigated in further study.

The PCA reveals clear sensory differentiation among sample groups. The Sauvignon group may be better suited for consumers preferring milder, more refreshing flavors due to its association with acidity and aqueousness. The Chardonnay group is more complex and diverse, with some samples being saltier or more fat, and others sharing similarities with the Sauvignon group. This fattiness could be brought about by barrel aging and could reflect a higher polysaccharide and glycerol content. The Viognier group appears intermediate but leans toward bitterness and umami, suggesting potential for more intense flavor profiles.

This sensory discrimination can inform product development strategies, such as targeting specific sensory descriptors for further investigation or reformulation.

## 5. Conclusions

This study demonstrates that trained panelists possess a statistically significant ability to discriminate between white wine grape varieties under controlled sensory evaluation conditions. Olfactory perceptions yielded higher recognition rates than just gustatory ones, with nine out of fourteen panelists showing a statistically significant performance. Although the paired *t*-test revealed only a marginally significant difference between olfactory and gustatory modalities, the overall trend and binomial test results underscore the dominant role of olfaction in varietal recognition.

The RATA sensory provided significant varietal effects observed for acidity, fattiness, bitterness, and alcohol, suggesting that these attributes contribute to the perceived taste typicality of the wines.

Principal Component Analysis further revealed distinct sensory profiles among the varieties, with Sauvignon B characterized by high acidity and aqueousness, Chardonnay by greater fattiness, and Viognier showing intermediate and variable attributes, with slightly more perceived salty. These findings indicate that certain grape varieties may possess specific taste-active compounds that contribute to varietal typicality and enable partial recognition through taste alone.

Overall, the results confirmed the already known importance of olfactory perception in varietal white wines recognition and provided new innovative results of taste attributes to define sensory taste groups for these wines.

Further research is needed to define the molecular family or physico-chemical attributes that can discriminate wines varieties. A full metabolomic study is necessary to analyze thousands of molecules and will be undertaken to discover the taste active compounds.

## Figures and Tables

**Table 1:** Percentage of correct answers (question: is this a Chardonnay or not?) and associated *p*-value (* if *p* ≤ 0.05, ** if *p* ≤ 0.01, *** if *p* ≤ 0.001) for each varietal wine (C: Chardonnay, S: Sauvignon B, V: Viognier).

		**C1**	**C2**	**C3**	**C4**	**C5**	**C6**	**C7**	**C8**	**C9**	**C10**
Taste	% of correct answers	50	50	64	43	50	50	71	43	57	57
	*p*-value	0.395	0.395	0.090	0.605	0.395	0.395	0.029	0.605	0.212	0.212
								*			
Smell	% of correct answers	79	79	71	57	57	36	43	50	43	86
	*p*-value	0.006	0.006	0.029	0.212	0.212	0.788	0.605	0.395	0.605	0.001
		**	**	*							***
Total	% of correct answers	64	64	68	50	54	43	57	46	50	71
	*p*-value	0.044	0.044	0.018	0.425	0.286	0.714	0.172	0.575	0.425	0.006
		*	*	*							**
		**S1**	**S2**	**S3**	**S4**	**S5**	**V1**	**V2**	**V3**	**V4**	**V5**
Taste	% of correct answers	57	64	71	93	71	79	36	71	50	50
	*p*-value	0.212	0.090	0.029	0.000	0.029	0.006	0.788	0.029	0.395	0.395
				*	***	*	**		*		
Smell	% of correct answers	93	36	86	100	64	57	86	86	93	86
	*p*-value	0.000	0.788	0.001	0.000	0.090	0.212	0.001	0.001	0.000	0.001
		***		***	***			***	***	***	***
Total	% of correct answers	75	50	79	96	68	68	61	79	71	68
	*p*-value	0.002	0.425	0.000	0.000	0.018	0.018	0.092	0.000	0.006	0.018
		**		***	***	*	*		***	**	*

**Table 2:** Analysis of variance for taste descriptors by panelistand variety (* if *p* ≤ 0.05, *** if *p* ≤ 0.001).

	Acidity	Alcohol	Bitterness	Aqueous	Fat	Sweetness	Short	Long	Salty	Umami
Panelist	<0.0001 ***	<0.0001 ***	<0.0001 ***	<0.0001 ***	<0.0001 ***	<0.0001 ***	<0.0001 ***	<0.0001 ***	<0.0001 ***	<0.0001 ***
Variety	<0.0001 ***	0.021 *	0.038 *	0.115	<0.0001 ***	0.166	0.342	0.747	0.063	0.343

**Table 3:** Tukey test results for comparisons of sensory profiles between the varieties (* if *p* ≤ 0.05, *** if *p* ≤ 0.001).

	Acidity ***			Alcohol *			Fat ***			Bitterness *			Salty		
Chardonnay	1.7		B	1.3	A		1.2	A		1.3	A		0.9	A	
Sauvignon	2.6	A		0.9		B	0.5		B	0.9		B	0.7	A	B
Viognier	1.7		B	1.1	A	B	1.4	A		1.0	A	B	0.6		B

## Data Availability

The original contributions presented in this study are included in the article. Further inquiries can be directed to the corresponding author.

## Abbreviations

The following abbreviations are used in this manuscript:

RATA	Rate All That Apply
RGPD	Regulation on the protection of personal data
S	Sauvignon Blanc
C	Chardonnay
V	Viognier

## Appendix A. Training Standards for Sensory Analysis

**Table A1:** List of standards used for RATA Analysis.

Descriptor	Product	Concentration
Acid	Tartaric acid	1.25 g/L
Bitterness	Caffeine	1 g/L
Aqueous	Water	200 mL/L
Alcohol	Absolute ethanol	40 mL/L
Sweetness	Rectified concentrated must	5 mL/L
Fat	Cellulose gum	20 g/L
Salty	KCl salt	300 mg/L
Umami	Monosodium glutamate	3 g/L

## Appendix B. Information on the Wines Used

**Table A2:** Percentage of alcohol and information on winemaking.

Code	Alcohol (%)	Information on Winemaking
C1	13	Fermentation in stainless steel tanks, stirred, 5–6 months ageing in barrels
C2	13.5	Fermented in barrels, stirred, 8–9 months ageing in oak barrels
C3	12.5	Fermentation in barrels, 3 months ageing in barrels
C4	13	Fermentation in stainless steel tanks, partially aged (1/3) in barrels for 6 months
C5	12.5	Temperature-controlled fermentation in stainless steel tanks
C6	14	15-day fermentation in tanks, stirred, ageing on the lees
C7	13	Fermentation in stainless steel tanks
C8	13	Low temperature fermentation in stainless steel tanks
C9	13	Old vines, ageing in barrels
C10	13	Low temperature fermentation in stainless steel tanks
S1	12.5	Low temperature fermentation in stainless steel tanks
S2	13	Low temperature fermentation in stainless steel tanks
S3	12	Fermentation in stainless steel tanks
S4	13	Fermentation in stainless steel tanks
S5	12.5	Temperature-controlled fermentation in stainless steel tanks
V1	13.5	Temperature-controlled fermentation in stainless steel tanks, ageing on fine lees for 6–9 months.
V2	13.5	15-day fermentation in stainless steel tanks
V3	13.5	Low-temperature fermentation in stainless steel tanks, partially aged (30%) in barrels
V4	13	Temperature-controlled fermentation in stainless steel tanks
V5	13.5	Temperature-controlled fermentation in stainless steel tanks
